# Higher dietary glycemic load is inversely associated with stress prevalence among Iranian adults

**DOI:** 10.1186/s12868-022-00713-z

**Published:** 2022-05-20

**Authors:** Ali Amirinejad, Mina Darand, Ian G. Davies, Mohsen Mazidi, Azadeh Nadjarzadeh, Masoud Mirzaei, Sayyed Saeid Khayyatzadeh

**Affiliations:** 1 Nutrition and Food Security Research Center, Shahid Sadoughi University of Medical Sciences, Shohadaye Gomnam BLD, ALEM Square, Yazd, Iran; 2 Department of Nutrition, School of Public Health, Shahid Sadoughi University of Medical Sciences, Yazd, Iran; 3 Department of Clinical Nutrition, School of Nutrition and Food Science, Isfahan University of Medical Sciences, Isfahan, Iran; 4 Food Security Research Center, Isfahan University of Medical Sciences, Isfahan, Iran; 5 Research Institute of Sport and Exercise Sciences, Liverpool John Moores University, Liverpool, UK; 6 Nuffield Department of Population Health, University of Oxford, Richard Doll Building, Old Road Campus, Oxford, OX3 7LF UK; 7 Yazd Cardiovascular Research Center, Non-Communicable Disease Institute, Shahid Sadoughi University of Medical Sciences, Yazd, Iran

**Keywords:** Anxiety, Depression, Dietary glycemic index, Dietary glycemic load, Stress

## Abstract

**Background:**

Psychological disorders including depression, anxiety, and stress comprise a huge public health problem. The aim of this cross-sectional study is to assess the relationship between dietary glycemic index (DGI) and glycemic load (DGL) and mental disorders.

**Method:**

Participants (n = 10,000) aged 20–69 were randomly selected from 200 clusters in Yazd from the recruitment phase of Yazd Health Study. The dietary intake of study participants was collected by a reliable and validated food frequency questionnaire consisting of 178 food items. DGI and DGL were calculated from the FFQ data using previously published reference values. To assess psychological disorders an Iranian validated short version of a self-reported questionnaire Depression Anxiety Stress Scales 21 was used.

**Results:**

There were no significant associations between DGI and DGL with odds of depression or anxiety in crude and adjusted models. However, individuals in the highest quartiles of DGL had the lowest odds of stress (OR: 0.69; 95% CI 0.47–1, P-trend = 0.023). This association remained significant after adjustment for potential confounding variables in model I (OR: 0.45; 95% CI 0.22–0.9, P-trend = 0.023), model II (OR: 0.46; 95% CI 0.22–0.96, P-trend = 0.039) and model III (OR: 0.46; 95% CI 0.22–0.96, P-trend = 0.042).

**Conclusion:**

In conclusion, consumption of foods with higher GL was associated with lower odds of stress; however, no significant association was found between DGI or DGL and risk of depression and anxiety. Performing further studies with longitudinal design is suggested to confirm these results.

## Introduction

Psychological disorders including depression, anxiety, and stress comprise a huge public health problem, affecting about 31% to 41% of the world population in 2020 [1]. The pathophysiology of mental disorders is complex, with genetic, biological, and environmental factors involved in its onset and progression [2]. The underlying mechanisms are not fully understood, which may explain the poor response (50%) of current pharmacotherapies [3].

Among the environmental factors, dietary intakes have long been demonstrated to be associated with mental disorders. For example, diets rich in fruits and vegetables have a general positive impact on mental health [4] whereas a western diet (characterized by high intake of fried food, processed meat, refined carbohydrate and confectionary) is associated with mental disorders [5]. The impact of dietary carbohydrate intake on health outcomes and disease has increasingly been in focus in recent years [6]. However, the link between the various aspects of carbohydrate and psychological health remains unclear. The effects of foods to increase blood glucose are different and this property is considered the glycemic index (GI), a quality rating of how individual foods raise blood sugar levels. In fact, GI is the increase in postprandial blood glucose after consumption of a specific carbohydrate portion of a food compared with glucose or white bread [7]. When we consume high-GI foods, blood glucose and subsequently insulin concentrations are rapidly increased, while smaller and slower elevation of postprandial blood glucose and insulin levels are observed following intake of low-GI foods. Refined grains such as white bread, rice, potatoes and sugary products are considered high-GI foods, whereas vegetables, whole grain and legumes are included in low-GI groups [8]. Dietary glycemic load (GL) is the product of a food’s GI and total available carbohydrate content, and provides both the quality and quantity the of carbohydrates [9]. Thus, GL represents a more accurate view of a food’s real-life effects on postprandial glucose and insulin response.

The prevalence of psychological disorders is much higher in patients with diabetes [10] and some studies show a link between DGI and DGL with psychological disorders [11–13], suggesting glucose metabolism might play a mechanistic role. Indeed, high GI and GL foods provoke insulin secretion leading to subsequent hypoglycemia which impact on the nervous system and result in psychological disorders [14]. However, increased insulin levels from high GI/GL diets may facilitate delivery of tryptophan in the brain and increase the synthesis of serotonin, a neurotransmitter associated with mood improvement [15]. On the other hand, most of fruits and vegetables which are the good source of dietary fiber and several antioxidant compound such as phytochemical are classified in the medium and low GI foods [16]. These nutrients had positive effects on mental disorders in previous studies [17, 18].

Totally, the literature in this field is equivocal, probably due to study design, sample size, duration, and other components of the diet that may explain this inconsistency [19–23]. Therefore, due to the necessity of conducting more high-quality research, we investigated the association of DGI and DGL with depression, anxiety, and stress in a large Iranian population.

## Materials and methods

### Study population

We used data from the enrollment phase of Yazd Health Study (YaHS) conducted from September 2014 to December 2015. YaHS is a prospective cohort study which has been conducted on 10,000 participants aged 20–70 years since 2014. The participants were randomly selected from 200 clusters in Yazd Greater Area. The profile and details of this study were published elsewhere [24]. Written informed consent was taken from all participants. The research was approved by the Ethics Committee of Shahid Sadoughi University of Medical Sciences, Yazd, Iran (Ethic code: 931188). Data regarding current and history of chronic diseases, smoking status, socio-demographic characteristics including age, gender, marital status and education level were obtained by interview and standard questionnaires. Participants were excluded on the following: under or over estimation of energy intake (total daily energy intake less than 800 or higher than 6500 kcal/day), pregnancy, following a special diet and taking antidepressants. After exclusion, 7384 participants (3673 men and 3711women) participated in the study.

### Dietary assessment

Dietary intake of study participants was collected by a reliable and validated food frequency questionnaire (FFQ) consisting of 178 food items designed for an Iranian population [25]. FFQs were completed during face-to-face interviews and reported dietary intakes in household measures were converted to grams and entered to the Nutritionist IV software (First Databank Inc., Hearst Corp., San Bruno, CA, USA). To calculate daily nutrient intake values for each participant, the US Department of Agriculture’s (USDA) national nutrient databank was used [26].

### Dietary glycemic index and load calculation

The total DGI was calculated based on the following formula: $\sum {{{\left( {{\text{GIa}} \times {\text{available}}\;{\text{carbohydrate}}} \right)} \mathord{\left/ {\vphantom {{\left( {{\text{GIa}} \times {\text{available}}\;{\text{carbohydrate}}} \right)} {{\text{total}}\;{\text{available}}\;{\text{carbohydrate}}}}} \right. \kern-\nulldelimiterspace} {{\text{total}}\;{\text{available}}\;{\text{carbohydrate}}}}} .$ The available carbohydrate content of foods was calculated as total carbohydrate minus fiber [27]. The total carbohydrate and fiber contents of foods were adapted from the US Department of Agriculture food-composition table [28]. Food items with low carbohydrate content (less than 3.5 g available carbohydrate per serving) like tomatoes, pickles, cabbage, cucumbers, lettuce, cheese, sausages, eggs, mayonnaise were excluded because GI values of these foods could not be tested [29]. Of the 178 and 121 food and beverage items in the FFQs of YaHS-TAMYS study, 43 (24.1%) and 32 (24.4%) items contained less than 3.5 g available carbohydrate/serving, respectively. Eventually, the calculation of GI was carried out based on the remaining 135 items in the YaHS-TAMYS. GI values for 108 food items in the YaHS-TAMYS study were adapted from the international references [16, 30]. for Iranian specific foods not covered in the international tables (6 items), the Iranian GI tables were used [31], because the GI of all food items was not covered. GI values for the remaining food items which were not available, neither in Iranian nor on the international tables, such as some traditional sweets and desserts, were gained based on physically and chemically similar foods. For example, the GI value for gaz, a traditional food item highly consumed in Yazd city and mainly made of flour, almonds, and sugar, was considered to be the same as sugar. When more than one GI value from a different brand was available, the mean GI value was applied (e.g. rice and dates). The GIs of mixed meals were obtained based on GI values of each of the meal’s components [28]. For all derived GI values, glucose was used as the reference food. Finally, DGL was calculated by multiplying the dietary GI by the total daily available carbohydrate intake and dividing the result by 100 [28].

### Anthropometric measurements

Body weight was measured using a portable digital scale analyzer with an accuracy of 0.1 kg. The participants stood in the middle of the scale, wearing the minimum possible clothing. Height was also measured in a standing position, while barefoot with the head placed in the Frankfurt position. Body Mass Index (BMI) was calculated, body weight (kg)/height (m^2^).

### Psychological health assessment

An Iranian validated short version of the self-report questionnaire for depression, anxiety and stress (DASS 21) consisting of seven items per subscale was used [32]. The individuals read each statement and recorded their reply according to a 4-point Likert scale ranging from 0 (does not apply to me at all) to 3 (applies to me very much or most of the time). The scores were summed for items of each scale. As the long form of DASS has 42 items, we multiplied the final score of each scale by two. The individuals were considered to have depression, anxiety, and stress if they obtain total scores of ≥ 10, ≥ 8 and ≥ 15, respectively.

### Statistical analysis

The normality of data was assessed using the Kolmogorov–Smirnov test. Continuous and categorical variables were compared across quartiles of DGI and DGL using analysis of variance (ANOVA) and chi-square tests respectively. The differences between nutrients and dietary intake of each quartile were assessed using a Bonferroni post-hoc analysis. Logistic regression was applied to evaluate the relationship between DGI and DGL with psychological disorders in crude and adjusted models. Model I was adjusted for age, gender, and total energy intake. In model II, marital status, smoking, education level, employment status, salt intake, multi-vitamins use, diabetes, and hypertension were additionally adjusted. Further adjustment was for BMI in the final model. *P*-values < 0.05 were considered statistically significant. To analyze the data, the statistical Package for Social Sciences (SPSS) (version 23.0, SPSS Inc., Chicago, Illinois, USA) was used.

## Results

Overall, 7384 participants were included (3673 men and 3711 women). The prevalence of psychological disorders did not significantly differ among quartiles of DGI and DGL. BMI, marital status, education level, multi-vitamin supplement use, and hypertension were significantly different among quartiles of DGI. Furthermore, significant differences were seen for age, physical activity, marital status, gender, employment status, education level, multi-vitamin supplement use, hypertension, and diabetes across quartiles of DGL (Table 1).

**Table 1:** General characteristics of participants across quartiles of GI and GL

Variable	GI						GL				
	Total	Q1	Q2	Q3	Q4	P-value^a^	Q1	Q2	Q3	Q4	P-value
Depression, yes (%)	578 (8.1)^a^	148 (8.3)	142 (7.9)	130 (7.2)	158 (8.9)	0.326	158 (8.8)	153 (8.6)	132 (7.3)	135 (7.6)	0.252
Anxiety, yes (%)	754 (10.5)	186 (10.4)	186 (10.3)	170 (9.5)	212 (11.9)	0.113	200 (11.1)	190 (10.7)	183 (10.1)	181 (10.2)	0.710
Stress, yes (%)	238 (3.3)	58 (3.2)	65 (3.6)	54 (3)	61 (3.4)	0.776	68 (3.8)	69 (3.9)	54 (3)	47 (2.7)	0.106
BMI (kg/m^2^)											
< 18.5	247 (3.3)	61 (3.2)	63 (3.3)	75 (4)	48 (2.5)	0.018	57 (3)	55 (2.9)	73 (3.9)	62 (3.3)	0.112
18.5–24.9	2339 (30.9)	595 (31.4)	618 (32.6)	586 (30.9)	540 (28.5)		577 (30.5)	630 (33.3)	577 (30.5)	555 (29.3)	
> 25	4988 (65.9)	1237 (65.3)	1213 (64)	1233 (65.1)	1305 (68.9)		1259 (66.5)	1209 (63.8)	1244 (65.7)	1276 (67.4)	
Age (%)											
20–29 years	1586 (21.5)	419 (22.7)	404 (21.8)	404 (217)	359 (19.6)	0.052	322 (17.4)	404 (22)	433 (23.4)	427 (23.2)	< 0.01
30–39 years	1598 (20.8)	383 (20.8)	395 (21.4)	380 (20.4)	440 (24)		324 (17.5)	405 (22)	429 (23.1)	440 (23.9)	
40–49 years	1586 (21.5)	387 (21)	413 (22.3)	393 (21.1)	393 (21.4)		404 (21.8)	397 (21.6)	399 (21.5)	386 (21)	
50–59 years	1402 (19)	377 (20.4)	347 (18.8)	348 (18.7)	330 (18)		392 (21.2)	341 (18.5)	323 (17.4)	346 (18.8)	
60–69 years	1215 (16.4)	279 (15.1)	290 (15.7)	334 (18)	312 (17)		409 (22.1)	293 (15.9)	270 (14.6)	243 (13.2)	
*Physical activity* (Met_min/week)	901.1^b^ ± 905.1	890.8 ± 883.1	894.5 ± 910.5	877.5 ± 875	942.4 ± 950.3	0.156	826.6 ± 876.7	896.8 ± 896.4	932.8 ± 903	949 ± 939.7	< 0.01
Marriage (%)											
Single	856 (11.6)	232 (12.6)	225 (12.2)	206 (11.1)	193 (10.6)	0.019	178 (9.6)	224 (12.2)	220 (11.9)	234 (12.8)	0.001
Married	6264 (85.1)	1575 (85.6)	1567 (84.8)	1570 (85.9)	1570 (85.9)		1587 (86)	1541 (84.2)	1585 (85.6)	1551 (84.6)	
Widowed or divorced	240 (3.3)	61 (3.3)	40 (2.2)	75 (4.1)	64 (3.5)		80 (4.3)	65 (3.6)	46 (2.5)	49 (2.7)	
Smoking status (%)											
Never smoker	6328 (87.8)	1582 (87.6)	1576 (87)	1617 (89)	1553 (87.5)	0.496	1609 (88.4)	1567 (86.9)	1587 (87.7)	1565 (88.3)	0.520
Current smoker	766 (10.6)	194 (10.7)	207 (11.4)	177 (9.7)	188 (10.6)		190 (10.4)	200 (11.1)	192 (10.6)	184 (10.4)	
Ex_smoker	113 (1.6)	29 (1.6)	29 (1.6)	22 (1.2)	33 (1.9)		22 (1.2)	36 (2)	31 (1.7)	24 (1.4)	
Gender, male (%)	3673 (49.7)	909 (49.1)	927 (50.2)	917 (49.5)	920 (50.2)	0.877	831 (44.8)	923 (50.3)	967 (52)	952 (51.9)	< 0.01
Job (%)											
Unemployed	1415 (19.5)	353 (19.6)	325 (17.9)	357 (19.6)	380 (21.1)	0.336	358 (19.8)	359 (19.9)	306 (16.8)	392 (21.7)	< 0.01
Government employee	3537 (48.8)	898 (49.8)	898 (49.5)	904 (49.6)	837 (46.5)		960 (53.1)	898 (49.7)	876 (48.1)	803 (44.4)	
Manual worker	247 (3.4)	60 (3.3)	66 (3.6)	56 (3.1)	65 (3.6)		61 (3.4)	53 (2.9)	67 (3.7)	66 (3.6)	
Freelance job	2046 (28.2)	492 (27.3)	530 (29.1)	505 (27.7)	519 (28.8)		428 (23.7)	497 (27.5)	572 (31.4)	549 (30.3)	
Education (%)											
Illiterate	1776 (24.1)	438 (23.7)	418 (22.7)	500 (27)	420 (23.1)	0.004	562 (30.5)	401 (21.8)	406 (22)	407 (22.3)	< 0.01
Middle school	2095 (28.5)	501 (27.2)	508 (27.6)	514 (27.7)	527 (31.4)		527 (28.6)	549 (29.9)	494 (26.7)	525 (28.7)	
Diploma	2289 (31.1)	611 (33.1)	590 (32)	553 (29.8)	535 (29.4)		520 (28.2)	557 (30.3)	629 (34)	583 (31.9)	
Bachelors degree	995 (13.5)	253 (13.7)	261 (14.2)	204 (12.9)	241 (13.2)		201 (10.9)	270 (14.7)	260 (14.1)	264 (14.4)	
Master and doctor	205 (2.8)	42 (2.3)	65 (3.5)	47 (2.5)	51 (2.8)		35 (1.9)	62 (3.4)	59 (3.2)	49 (2.7)	
Multi*-*vitamins supplement (%)											
Never	6407 (86.1)	1727 (94.3)	1715 (91.9)	1619 (86.4)	1346 (72.1)	> 0.01	1741 (94.2)	1727 (93.7)	1691 (90)	1248 (66.8)	< 0.01
1–3/month	482 (6.5)	44 (2.4)	67 (3.6)	95 (5.1)	276 (14.8)		34 (1.8)	37 (2)	64 (3.4)	347 (18.6)	
Minimal once a week	549 (7.4)	60 (3.3)	85 (4.6)	159 (8.5)	245 (13.1)		73 (4)	79 (4.3)	124 (6.6)	273 (14.6)	
Diabetes, yes (%)	912 (12)	233 (12.3)	209 (11)	244 (12.9)	226 (11.9)	0.358	313 (16.5)	229 (12.1)	194 (10.2)	176 (9.3)	< 0.01
Hypertension, yes (%)	1135 (15)	280 (14.8)	280 (14.8)	321 (16.9)	254 (13.4)	0.023	350 (18.5)	286 (14.1)	277 (14.6)	240 (12.7)	< 0.01

*GI* glycemic index, *GL* glycemic load

^a^Obtained from χ^2^ test and one-way anova for categorical and continuous variables, respectively

^b^Data are presented as mean ± standard deviation (SD)

Dietary nutrients and energy adjusted food groups are presented in Table 2. The one-way ANOVA test followed by Bonferroni post-hoc analysis revealed significant differences between all dietary nutrients and food groups including, energy intake; percentage energy from protein, carbohydrate, and fat intake; cholesterol; saturated fatty acid; vitamin E; vitamin C; folic acid; magnesium; fruits; vegetables; red meat; fish, dairy; whole grains; refined grains; sugars; salt; legumes and nuts. Significant differences among the quartiles of DGI and DGL are shown with different letters “a” or “b”.

**Table 2:** Dietary intake of study participants among the quartiles of GI & GL

Quartiles of GI							Quartiles of GL				
	Total	Q1	Q2	Q3	Q4	*P*-value ^I^	Q1	Q2	Q3	Q4	*P*-value
Nutrients											
Energy intake (kcal)	2897 ± 1378	2438 ± 1061^a^	2864 ± 1351^b^	3634 ± 1351^b^	2897 ± 1378^b^	< 0.001	1646 ± 419.3^a^	2199 ± 494.1^b^	3019 ± 744.9^b^	4721 ± 1111^b^	< 0.001
Protein (% of total daily energy)	16.16 ± 0.04	16.82 ± 0.04^a^	16.73 ± 0.03	16.24 ± 0.04^b^	14.86 ± 0.04^b^	< 0.001	17.84 ± 0.04^a^	16.59 ± 0.03^b^	16.11 ± 0.03^b^	14.10 ± 0.04^b^	< 0.001
Carbohydrate (% of total daily energy)	56.12 ± 0.09	53.81 ± 0.08^a^	54.58 ± 0.08	56.00 ± 0.08^b^	60.09 ± 11.21^b^	< 0.001	52.55 ± 0.07^a^	55.40 ± 0.07^b^	55.87 ± 0.09^b^	60.67 ± 0.10^b^	< 0.001
Fat (% of total daily energy)	33.61 ± 0.09	32.90 ± 0.07^a^	32.67 ± 0.07	32.85 ± 0.07	0.36 ± 0.12^b^	< 0.001	33.24 ± 0.06^a^	31.53 ± 0.06^b^	33.38 ± 0.09	36.28 ± 0.11^b^	< 0.001
Cholesterol (mg)	394.8 ± 391.3	363.5 ± 396.1^a^	394.1 ± 326.6	391.1 ± 336.4	430.6 ± 483.4^b^	< 0.001	286.3 ± 322.9^a^	318.4 ± 206.4	432.7 ± 390.9^b^	542 ± 521.8^b^	< 0.001
SFA (g)	31.04 ± 18.36	27.13 ± 15.36^a^	29.58 ± 17.43^b^	30.48 ± 17.28^b^	36.98 ± 21.39^b^	< 0.001	19.56 ± 9.62^a^	24.3 ± 11.47^b^	32.47 ± 15.22^b^	47.8 ± 20.82^b^	< 0.001
Vitamin E (mg/day)	11.36 ± 11.66	11.92 ± 11.85	11.52 ± 11.80	10.98 ± 12.02	11.01 ± 10.92	0.038	7.70 ± 7.16^a^	9.55 ± 8.93^b^	12.72 ± 13.28^b^	15.45 ± 14.19^b^	< 0.001
Vitamin C (mg)	210.5 ± 188.7	196.2 ± 169^a^	218.3 ± 214.3^b^	219.2 ± 169.6^b^	208.5 ± 197.1	< 0.001	115.1 ± 54.12^a^	166.8 ± 92^b^	243.2 ± 148.4^b^	317.1 ± 292.6^b^	< 0.001
Folic acid (µg)	379.8 ± 228	354.6 ± 215.5^a^	361.9 ± 217.7	370.8 ± 216.6	432.1 ± 251.8^b^	< 0.001	230.7 ± 93.18^a^	300 ± 127.6^b^	410.8 ± 193.8^b^	577.8 ± 276.8^b^	< 0.001
Magnesium (mg)	339.3 ± 179.6	309.6 ± 155.2^a^	322.9 ± 166.2	336.2 ± 180.7^b^	339.3 ± 179.6^b^	< 0.001	199.7 ± 58.38^a^	262.8 ± 79.31^b^	359 ± 125.2^b^	535.7 ± 198.7^b^	< 0.001
Food groups (g/1000 kcal)											
Fruits	184.3 ± 138.4	205.6 ± 145.1^a^	202.3 ± 141.8	192.3 ± 130.3^b^	136.9 ± 124.1^b^	< 0.001	187.8 ± 109.9^a^	204.8 ± 128.1^b^	202.8 ± 151.3^b^	184.3 ± 138.4^b^	< 0.001
Vegetables	96.6 ± 77.29	112 ± 82.82^a^	104.1 ± 78.03^b^	97.77 ± 75.50^b^	72.76 ± 66.25^b^	< 0.001	119.6 ± 81.3^a^	104.7 ± 69.75^b^	96.78 ± 81.41^b^	65.57 ± 65.10^b^	< 0.001
Red meat	20.46 ± 18.37	22.60 ± 17.77^a^	24.60 ± 21.13^b^	20.12 ± 16.43^b^	14.51 ± 16.16^b^	< 0.001	25.29 ± 20.43^a^	23.65 ± 16.75^b^	20.90 ± 19.34^b^	11.99 ± 13.15^b^	< 0.001
Fish	7 ± 13.56	5.90 ± 11.50^a^	6.70 ± 12.80	7.48 ± 14.42^b^	7.91 ± 15.14^b^	< 0.001	5.85 ± 9.99^a^	5.65 ± 10.98	7.65 ± 14.93^b^	8.85 ± 16.90^b^	< 0.001
Dairy	89.84 ± 60.16	106.99 ± 76.74^a^	94.95 ± 53.32^b^	89.96 ± 53.41^b^	67.47 ± 45.57^b^	< 0.001	104.2 ± 53.30^a^	95.85 ± 52.83^b^	86.20 ± 59.35^b^	73.09 ± 69.35^b^	< 0.001
Legumes and nuts	25.20 ± 19.14	29.78 ± 25.53^a^	25.64 ± 18.63^b^	24.07 ± 14.99^b^	21.31 ± 14.35^b^	< 0.001	27.80 ± 18.12^a^	25.24 ± 18.01^b^	25.30 ± 20.19^b^	22.46 ± 19.80^b^	< 0.001
Whole grains	29.95 ± 27.33	26.68 ± 20.58^a^	32.32 ± 24.55^b^	33.81 ± 27.92^b^	27.01 ± 33.82	< 0.001	34.97 ± 24.53^a^	37.83 ± 28.35^b^	29.25 ± 28.89^b^	17.77 ± 22.65^b^	< 0.001
Refined grains	85.67 ± 58.18	126.70 ± 66.74^a^	93.05 ± 47.27^b^	67.95 ± 41.92^b^	55 ± 45.87^b^	< 0.001	100.5 ± 54^a^	102 ± 49.75	82.02 ± 59.34^b^	58.11 ± 58.05^b^	< 0.001
Sugars	12.63 ± 11.64	10.21 ± 7.58^a^	11.88 ± 9.05^b^	13.36 ± 11.61^b^	15.07 ± 15.98^b^	< 0.001	12.14 ± 9.27	12.74 ± 9.42	12.80 ± 12.58	12.84 ± 14.47	0.210
Salt	2.94 ± 3.68	3.21 ± 2.94^a^	3.22 ± 3.45	3.14 ± 3.89	2.21 ± 4.21^b^	< 0.001	4.24 ± 3.83^a^	3.61 ± 3.11^b^	2.41 ± 3.37^b^	1.52 ± 3.73^b^	< 0.001

*GI* glycemic index, *GL* glycemic load

^I^Obtained from one-way ANOVA followed by Bonferroni post-hoc test

^a^First quartile is considered as reference quartile

^b^Significant difference between quartile with reference quartile (P value < 0.05)

The association between DGI and DGL with the respective prevalence of depression, anxiety and stress in crude and adjusted models are presented in Table 3. There were no significant associations between DGI and DGL with odds of depression or anxiety in crude and adjusted models. However, individuals in the highest quartiles of DGL had the lowest odds of stress (*OR: 0.69; 95% CI 0.47–1, P-trend* = *0.023*). This association remained significant after adjustment for potential confounding variables in model I (*OR: 0.45; 95% CI 0.22–0.9, P-trend* = *0.023*), model II (*OR: 0.46; 95% CI 0.22–0.96, P-trend* = *0.039*) and model III (*OR: 0.46; 95% CI 0.22–0.96, P-trend* = *0.042*).

**Table 3:** Multivariable-adjusted ORs (and 95% CIs) for depression, anxiety and stress across quartiles of GI and GL

GI quartile						GL quartiles				
Variables	Q1	Q2	Q3	Q4	P-trend^‡^	Q1	Q2	Q3	Q4	P-trend
Depression [n (%)]										
Crude	1.00	0.94 (0.74–1.20)^†^	0.86 (0.67–1.10)	1.08 (0.85–1.36)	0.672	1.00	0.96 (0.75–1.22)	0.78 (0.61–1.01)	1.10 (0.87–1.39)	0.717
Adjusted model1^a^	1.00	0.96 (0.75–1.22)	0.87 (0.68–1.12)	1.16 (0.90–1.48)	0.403	1.00	1.01 (0.79–1.29)	0.86 (0.64–1.15)	0.97 (0.63–1.50)	0.546
Adjusted model2^b^	1.00	0.096 (0.74–1.24)	0.84 (0.64–1.10)	1.11 (0.85–1.46)	0.679	1.00	1.00 (0.77–1.30)	0.86 (0.63–1.18)	0.97 (0.61–1.56)	0.579
Adjusted model3^c^	1.00	0.096 (0.74–1.24)	0.84 (0.64–1.10)	1.12 (0.85–1.46)	0.653	1.00	1.00 (0.77–1.30)	0.86 (0.63–1.18)	0.97 (0.61–1.55)	0.604
Anxiety [n (%)]										
Crude	1.00	0.99 (0.80–1.22)	0.90 (0.72–1.12)	1.16 (0.94–1.43)	0.248	1.00	0.95 (0.77–1.17)	0.89 (0.72–1.10)	0.90 (0.73–1.12)	0.292
Adjusted model1^a^	1.00	1.00 (0.80–1.24)	0.91 (0.73–1.13)	1.22 (0.98–1.52)	0.157	1.00	0.96 (0.77–1.20)	0.90 (0.70–1.17)	0.94 (0.64–1.38)	0.568
Adjusted model2^b^	1.00	0.99 (0.78–1.25)	0.89 (0.70–1.13)	1.17 (0.92–1.49)	0.343	1.00	0.94 (0.74–1.20)	0.90 (0.68–1.19)	0.91 (0.60–1.38)	0.542
Adjusted model3^c^	1.00	0.99 (0.78–1.25)	0.89 (0.70–1.13)	0.94 (0.87–1.02)	0.323	1.00	0.94 (0.74–1.20)	0.90 (0.68–1.18)	0.91 (0.60–1.38)	0.573
Stress [n (%)]										
Crude	1.00	1.11 (0.77–1.60)	0.92 (0.63–1.35)	1.06 (0.73–1.53)	0.990	1.00	1.02 (0.72–1.44)	0.77 (0.54–1.11)	0.69 (0.47–1.00)	0.023
Adjusted model1^a^	1.00	1.13 (0.79–1.63)	0.93 (0.63–1.36)	1.12 (0.76–1.65)	0.839	1.00	0.97 (0.67–1.38)	0.64 (0.41–1.00)	0.45 (0.22–0.90)	0.023
Adjusted model2^b^	1.00	1.02 (0.69–1.51)	0.87 (0.57–1.31)	1.09 (0.072–1.65)	0.881	1.00	1.07 (0.68–1.50)	0.65 (0.40–1.05)	0.46 (0.22–0.96)	0.039
Adjusted model3^c^	1.00	1.02 (0.69–1.51)	0.87 (0.57–1.31)	1.10 (0.72–1.66)	0.888	1.00	0.98 (0.68–1.42)	0.72 (0.40–1.05)	0.46 (0.22–0.96)	0.042

*GI* glycemic index, *GL* glycemic load

^†^These values are odds ratios (95% CIs)

^‡^Obtained from logistic regression by considering quartiles of GI & GL as ordinal variable

^a^Adjusted for age, gender and total energy intake

^b^Additionally adjusted for physical activity, marital status, smoking, education, job status, multi-vitamins use, diabetes, hypertension

^c^Further adjustment BMI

## Discussion

This cross-sectional study assessed the association of dietary DGI and DGL with psychological disorders in an Iranian population. No significant association was observed between DGI and odds of depression, anxiety, and stress in crude and adjusted models. There was also no significant relationship between DGL and odds of depression or anxiety in crude and adjusted models; however, higher DGL was associated with lower odds of stress in all models.

A systematic review and meta-analysis by Salari-moghaddam et al. [33] was performed on studies with different designs to investigate the possible relation between DGI or DGL and depression. In line with our results, no significant association between DGI or DGL and odds of depression was found in cross-sectional studies. Although a positive association was observed between DGI and risk of depression in cohort studies, the number of studies was limited (n = 2) with significant heterogeneity between them [33].

Our results showed no significant association between DGI or DGL and odds of anxiety. In agreement with the current study, Haghighatdoost et al., after adjusting for confounders (marital status, education, physical activity, smoking, dietary intakes, and BMI) showed similar findings [19]. Another study with a cross-over clinical trial design examined the effects of a high GL diet on mental health. Consuming 28 days of a high GL diet did not alter tension-anxiety compared to the diet with a low GL [34].

There was also no association between DGI and stress. However, in line with a previous study [19], being in the highest quartiles of DGL in our study was associated with lower odds of stress. This inverse association can be attributed to the effects of sugar-rich foods on the hypothalamic–pituitary–adrenal (HPA) axis. Under stressful conditions, the HPA releases stress hormones (corticosteroids) that increase the desire for sugar rich foods that provide inhibitory feedback on the hypothalamus. Therefore, participants of the higher quartiles of DGL consumed more sugar-rich foods, which can lower stress by decreasing HPA axis activity [35, 36]. Furthermore, serotonin is a neurotransmitter that has an essential role in mood regulation. Foods with high GL induce insulin secretion which facilitates tryptophan uptake and serotonin production in the brain and thereby lowers stress [15]. However, it must be kept in mind that long-term consumption of food with high GL leads to blood glucose fluctuations and increase the risk of diabetes [37]. Thus, caution should be taken to account when interpreting these results since several studies reported that patients with diabetes have a higher risk of mental disorders compared to healthy people [38–40].

Besides the potential strengths of our study such as a large sample size covering both urban and rural areas, recruitment of well-trained interviewers, using a comprehensive and validated FFQ for evaluating dietary intake, and controlling for possible confounders, there are some limitations. First, the cross-sectional data of this study prevents any inference of causality between DGL and stress. Second, no biochemical measures were assessed in our study, which might limit the detection of patients with chronic disease. Furthermore, although the FFQ used in this study was validated for carbohydrate and intake of other nutrients, the validation for DGI or DGL was not performed. Thus, the observed association may be real or related to the quality of the questionnaire. Also, due to the absence of a reliable Iranian food composition table the USDA food-nutrient database was used, which is another limitation of this study. Finally, residual confounding from unknown or unmeasured variables could affect our results.

## Conclusion

In summary, an inverse association was found between DGL and likelihood of stress among Iranian adults. However, considering this point that long-term consumption of food with high GL may increase the risk of chronic diseases especially obesity and diabetes, we can not recommend high glycemic load dietary sources to the general population. Therefore, to clarify the effects of DGL or DGI on psychological profiles, further longitudinal studies such as randomized controlled trials are needed in at-risk populations.

## Data Availability

The data and materials of the current study are available from the corresponding author on reasonable request.
